# Evidence of Carbon Fixation Pathway in a Bacterium from Candidate Phylum SBR1093 Revealed with Genomic Analysis

**DOI:** 10.1371/journal.pone.0109571

**Published:** 2014-10-13

**Authors:** Zhiping Wang, Feng Guo, Lili Liu, Tong Zhang

**Affiliations:** 1 Environmental Biotechnology Laboratory, The University of Hong Kong, Hong Kong; 2 School of Environmental Science and Engineering, Shanghai Jiao Tong University, Shanghai, China; 3 State Environmental Protection Key Laboratory of Environmental Risk Assessment and Control on Chemical Process, East China University of Science and Technology, Shanghai, China; Wilfrid Laurier University, Canada

## Abstract

Autotrophic CO_2_ fixation is the most important biotransformation process in the biosphere. Research focusing on the diversity and distribution of relevant autotrophs is significant to our comprehension of the biosphere. In this study, a draft genome of a bacterium from candidate phylum SBR1093 was reconstructed with the metagenome of an industrial activated sludge. Based on comparative genomics, this autotrophy may occur via a newly discovered carbon fixation path, the hydroxypropionate-hydroxybutyrate (HPHB) cycle, which was demonstrated in a previous work to be uniquely possessed by some genera from *Archaea*. This bacterium possesses all of the thirteen enzymes required for the HPHB cycle; these enzymes share 30∼50% identity with those in the autotrophic species of *Archaea* that undergo the HPHB cycle and 30∼80% identity with the corresponding enzymes of the mixotrophic species within *Bradyrhizobiaceae*. Thus, this bacterium might have an autotrophic growth mode in certain conditions. A phylogenetic analysis based on the 16S rRNA gene reveals that the phylotypes within candidate phylum SBR1093 are primarily clustered into 5 clades with a shallow branching pattern. This bacterium is clustered with phylotypes from organically contaminated environments, implying a demand for organics in heterotrophic metabolism. Considering the types of regulators, such as FnR, Fur, and ArsR, this bacterium might be a facultative aerobic mixotroph with potential multi-antibiotic and heavy metal resistances. This is the first report on *Bacteria* that may perform potential carbon fixation via the HPHB cycle, thus may expand our knowledge of the distribution and importance of the HPHB cycle in the biosphere.

## Introduction

As the most diverse and abundant cellular life forms in the biosphere, microorganisms play key roles in nearly all biogeochemical processes. However, most microorganisms are not available in pure cultures and can only be detected with culture-independent molecular surveys, which greatly inhibits our comprehension of their roles in ecological and biogeochemical processes. The genomic sequencing of these microorganisms is significant in the construction of blueprints for evolutionary and metabolic diversity [1]. With advances in next generation sequencing (NGS) and bioinformatics, draft genomes of uncultured bacteria can be reconstructed from various complex environmental samples via single-cell genome sequencing [2] or genome binning [3]. Therefore, metabolic deductions and evolutionary analyses can be performed based on the reconstructed genomes and comparative genomics [4], which may greatly expand our understanding of microbial metabolism and its potential role in ecology and biogeochemistry.

SBR 1093 was established as a candidate phylum using several 16S rRNA gene clones in phosphate-removing activated sludge from a sequencing batch reactor [5] that was supplied with sodium acetate for phosphate removal. Thereafter, they were continuously detected in an industrial wastewater treatment system receiving low-molecular-weight organic acids and short-chain alcohols [6], activated sludge from coking wastewater treatment, chlorinated hydrocarbon-contaminated soil and hydrocarbon-contaminated soil [7]. All of these environments were associated with short-chain fatty acids, which implied that the bacteria within this candidate phylum may proliferate effectively with short-chain fatty acids. In addition to the contaminated environment, 16S rRNA clones within candidate phylum SBR1093 were also detected in samples from ocean environments, such as ocean crust from the East Pacific Rise [8], polymetallic nodules and the surrounding sediments, oceanic surface sediment [9], sponges [10], etc. Considering these specific niches, deficiency of light, O_2_ and organics, the most probable metabolism for these bacteria may be chemoautotrophy rather than heterotrophy. This is consistent with a report on a stalactite microbial community found in a desert cave [11] in which SBR1093-like 16S rRNA gene sequences comprised up to 10% of the total bacterial 16S rRNA gene sequences. Thus far, the metabolism of bacteria within candidate phylum SBR1093 remains elusive because there are no available pure cultures or enrichments from experiments or genomes. Because their abundance in the known microbial community is very low (less than 1% [11]), the metabolism of SBR1093 in these artificial and biogeochemical processes is difficult to deduce. Therefore, genome binning using the metagenome of a microbial community enriched with a member from this phylum could shed light on its metabolic properties and ecological functions.

As opposed to microbial communities in municipal wastewater treatment plants, which are fed with a mixture of natural organics and dominated by bacteria within *Proteobacteria*, *Bacteroidetes*, *Actinobacteria*, etc. [12], those in industrial wastewater treatment plants show unique populations in each plant [13]. Shaped by the specific substrates and physical-chemical conditions, microbial communities in industrial wastewater treatment plants are often enriched with uncultured microorganisms with specific metabolisms [14], and their metabolisms are associated with the biotransformation and biodegradation of specific substrates. Considering their relatively high abundance in these systems, draft genomes of the dominant populations could be reconstructed via the genome binning of the metagenome [15], [16] in an attempt to elucidate their physiological and ecological functions in the microbial community (as well as their taxonomy) [17]. Based on a survey of 454 pyrosequencing for the microbial community pyrosequencings in industrial-activated sludge (data not shown here), a bacterium of candidate phylum SBR1093 was enriched in a full industrial wastewater treatment plant (WWTP), which fed with morpholine distilling-wastewater and performed an alternating anoxic/aerobic process. The objective of this study is to reconstruct the draft genome of a bacterium from candidate phylum SBR1093 with the metagenome of activated sludge from this WWTP. This may shed light on its taxonomic identity, metabolic properties and ecological role, thus be helpful in determining potential conditions for its cultivation and isolation.

## Materials and Methods

### Sample collection and DNA extraction

Activated sludge samples were collected from a local industrial wastewater treatment plant: two samples from anoxic and aerobic tanks, respectively, which were fed with morpholine distilling-wastewater and that operated in alternate anoxic/aerobic processes (There is no specific permission required for the collection of sludge samples. This sampling site is located at the Shanghai Industrial Park, N31.01, E121.41, and the field studies did not involve endangered or protected species.). The collected samples were fixed onsite with absolute ethanol at a volume ratio of 1∶1, and then transported in an icebox and stored at −20°C prior to DNA extraction. For the DNA extraction, the microbial cells in the samples were collected after centrifugation and washed twice with phosphate-buffered solution (PBS). The DNA extraction was performed according to the protocol of the FastDNA SPIN Kit for soil (Qbiogene Inc., CA, USA), which was verified as the most suitable method to extract DNA from the activated sludge samples [18].

### Metagenomic sequencing

With the extracted DNA, libraries with insert sizes of 200 bp and 800 bp were constructed for each sample according to the manufacturer’s instructions (Illumina, San Diego, USA). Then, the metagenomic sequencing was performed with an Illumina HiSeq 2000 Platform (Illumina, San Diego, USA) using the 101 bp paired-end (PE101) strategy (BGI, Shenzhen, China). With a 2/5 Illumina sequencing run for the 200-bp library and a 1/5 sequencing run for the 800-bp library, approximately 101 and 35 million sequencing reads (100 bp) were obtained, respectively. Raw reads containing any ambiguous bases or those with an average quality score lower than 20 were removed prior to the following analysis.

### De novo assembly

The filtered reads were imported into the CLC genomic workbench (version 4.9), and the paired-end sequences were used for the following *de novo* assembly in the CLC genomic workbench. The K parameter (k-mer size) was set to 51 (half of the PE sequencing length) during the assembly. Only contigs longer than 500 bp were output as well as the corresponding mapping reads for further analysis. More than 50% of the reads were assembled into contigs >500 bp (98,505 contigs), with a maximum length of 349,894 bp. As a test to examine the potential errors in the assembly, the coverage consistence of the assembled contigs was checked according to the previous report [15].

### Genome binning

Genome binning was performed according to the previous work [19], based on a plot of coverage and GC ratio of contigs, including PE-tracking and reassembly, which was further refined with Metacluster 4.0 [20]. Then, the integrity and redundancy of the binning draft genome were assessed via the comparison of essential single copy genes (ESCGs) of most organisms in the domain *Bacteria* [15], [19].

### Gene annotation and comparison

Open reading frames (ORFs) were predicted online with MetaGeneMark [21], and the deduced amino acid (AA) sequences were obtained for BLASTp against the NCBI nr database (released on July 18, 2013) with an E-value of 10^−5^ and minimum alignment of 50 AA, respectively [22]. The results were taxonomically assigned with the lowest common ancestor (LCA) algorithm and functionally annotated based on KEGG using MEGAN 4.0 [23]. A Pfam search with an E-value of 10^−5^ was performed based on the Hidden Markol Model and against PfamA database version 26.0 [24], which could also be used for the comparison of gene clusters and verification of MEGAN annotation.

## Results and Discussion

### Genome binning and completeness assessment

**Figure 1:**
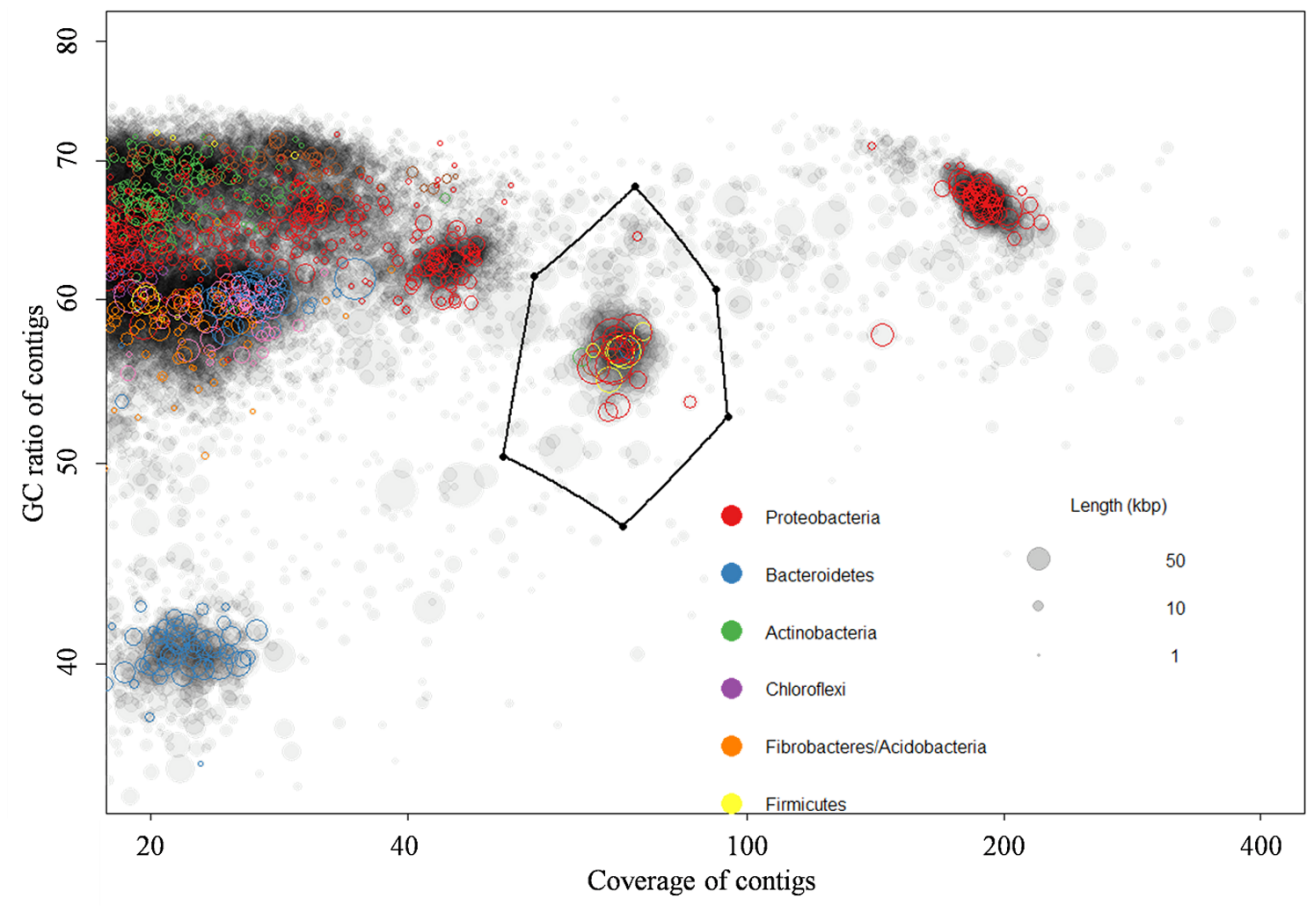
Genome binning of the dominant population with a plot of assembly contigs (based on coverage versus GC ratio). The circles represent the contigs with the size of the square root of their length. Clusters of contigs with similar color present potential genome bins, and contigs cluster with a coverage of approximately 80 (enclosed with black line) were collected for genome binning in this study.

A draft genome containing 94 scaffolds with a total size of 3,099,643 bp with GC contents of 56.4% was reconstructed (Figure 1). According to the prediction of MetaGeneMark, 3,228 ORFs were presented and 3,037 were in full length with the sole initiator and terminator, implying that the *de novo* assembly was accurately performed and that only the ORFs at the ends of the contigs were incomplete. Considering the functional assignment, 2,532 ORFs shared a mean similarity of 51.2% with the known enzymes in the nr-database (released on July 18, 2013), which is nearly at full align length (with a mean cover ratio of 0.87). All of the 40 universally occurring clusters of orthologous groups (COGs) [25] and tRNAs for all 20 amino acids are presented in this draft genome (Table S1 in File S1), which implies that it is near completeness. Additionally, based on the Hidden Markov Model (HMM) search, 102 unique ESCGs were found in this draft genome (Table S2 in File S1), indicating a completeness >96% [26]. Among the four suspected repetitive ESCGs, TIGR00436 and PF00750 are not always a single copy gene [19], and TIGR02350 is hit with the duplicate genes located within contig_838, implying no contamination from other bacteria. The only suspected duplicate ESCGs (PF00162) are distributed in different contigs and in best hit on the NCBI web to sequences in heterotrophic *Anoxybacillus* sp. (YP002316858) and autotrophic *Nitrososphaera* sp. (YP006862459), respectively. These two sequences are assigned as glyceraldehyde 3-phosphate dehydrogenase and triosephosphate isomerase, which are responsible for glycolysis and gluconeogenesis, respectively, and thus may coexist in an autotrophic or mixotrophic bacterium (consistent with the following metabolism analysis). In summary, this draft genome has no verifiable contamination from other bacteria.

### Phylogenetic and biogeographic characterization

**Figure 2:**
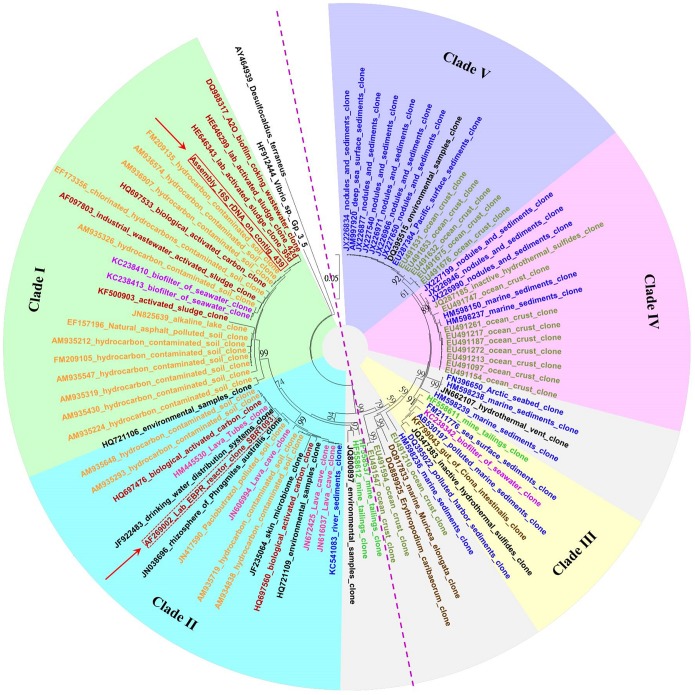
Phylogeny of phylotypes affiliated with the candidate phylum SBR1093. This phylogenetic tree is constructed with 16S rRNA gene sequences based on the neighbor-joining method with Jukes and Cantor distances. The main clades with nodes supported by a bootstrap value of >50% are labeled and marked with different background colors (Clade I green, Clade II blue, Clade III yellow, Clade IV pink and Clade V purple). The phylotypes derived from different sources are labeled with the following: dark red, activated sludge; orange, soil; blue, sediments; dark green, ocean crust; pink, lava; purple, seawater; green, mine tailings; brown, marine organisms; black, others. The phylotype SRB1093 HKSP obtained in this study is enclosed with a solid red line, whereas the first reported phylotype is enclosed with a red dashed line. The scale bar represents 0.05 nucleotide substitutions per site.

This draft genome contains a complete rRNA operon (16S, 5S and 23S, 3,379–8,941 bp) on contig_439, and the 16S rRNA gene (1,567 bp, 6,992–8,558 bp) is used for the taxonomic identification with BLASTn against NCBI and the Greengenes 16S rRNA gene database (released at May, 2013). The genome shares only 85.9% similarity with the 16S rRNA gene of pure culture *Vibrio* sp. Gp-3–5.1 (HF912444) but approximately 94.9% similarity with the first nominated SBR1093 sequence (AF269002) and 99.9% with the uncultured bacterium (HE646343). Therefore, this draft genome should represent a bacterium from candidate phylum SBR1093, named as SBR1093 HKSP. The neighbor-joining and maximum-likelihood phylogenetic tree of the 16S rRNA gene in this bacterium and strains from relative phyla revealed that the candidate phylum SBR1093 represented by this bacterium is close to *Proteobacteria* (Figure S1 in File S1). Additionally, as shown in the phylogenetic tree of this 16S rRNA gene and the reference sequences (Figure 2), phylotypes from candidate phylum SBR1093 are primarily divided into two subdivisions, terrestrial and marine. They are further clustered into 5 clades within which SBR1093 HKSP belongs to clade I. The shallow branching pattern within this phylum (the largest distance between these phylotypes is less than 0.12) implies that bacteria within SBR1093 are recently radiated [27]. It should be noted that some sequences named SBR1093 (GQ348518, 350258, 350948, AY907765, EF573230) are clustered with *Vibrio* sp. (HF912444) and should be excluded from this candidate phylum. Clades within this candidate phylum are distinguished clearly according to their biogeographic distributions, which are differed from dissolve oxygen, salinity, pH, as well as organic nutrients. Bacteria in clades I and II are primarily found in terrestrial environments associated with fertile organics, whereas those in clade IV and V are primarily found in barren marine environments. The 16S rRNA gene of this draft genome SBR1093 HKSP is clustered with phylotypes found in the activated sludge or contaminated environment (with similarity >99.9%), conditions with plenty of organics, thus implying the potential of organic metabolism (see details below).

In addition to the taxonomy of the 16S rRNA gene, proteins encoding in the genome may be another important resource in determining the phylogenetic position of an unknown bacterium. With the results of BLASTp checked against those in the nr-database, MEGAN may be used to classify these proteins using an LCA algorithm. Therefore, the ancestry of this bacterium can be speculated according to the phylogenetic relationship of proteins encoding in this draft genome. Of the 2,532 proteins in SBR1093 HKSP that have homologs with proteins in the nr-database, the largest section is clustered within the phylum level of *Proteobacteria* (n = 549), which is followed by *Firmicutes* (n = 70), *Cyanobacteria* (n = 53) and *Bacteroidetes* (n = 42) (Figure S2 in File S1). On the genus level, the most hits (n = 104) belong to *Geobacter* in *Deltaproteobacteria*, and the rests are evenly distributed among more than 50 genera. Therefore, candidate phylum SBR1093 represented by this bacterium may be close to *Proteobacteria* and *Firmicutes* but nonetheless separate from them, which is consistent with the taxonomy analysis of the 16S rRNA gene.

### Bacterial morphology and cell wall type

This SBR1093 bacterium might be rod-shape, for there is a complete set of genes for rod shape proteins identified in this draft genome (gene_1559–1563). Considering with the cell wall type, a complete set of genes responsible for peptidoglycan biosynthesis (gene_1066–1072) and outer membrane lipoprotein encoding (gene_41, 44, 411 and 2121) are identified, which are only present in Gram-negative bacteria, suggesting this bacterium might be Gram negative. During the life cycle, the bacterium may form spores in adverse conditions and germinate in more suitable conditions, because there is a complete set of genes for spore formation, maturation and germination (gene_1062, 1522 and 265).

### Primary metabolism

According to the KEGG annotation in MEGAN, approximately half of the predicted proteins (1,153 of the total 2,329) belong to metabolism, including 268 for carbohydrate metabolism, 223 for amino acid metabolism and 76 for lipid metabolism. The genes responsible for glycolysis/gluconeogenesis and the citrate cycle are complete in this draft genome, as well as a set for oxidative phosphorylation (Table S3 in File S1), indicating that this bacterium should be an aerobic heterotroph. However, two genes encoding the CRP/FNR family regulator and five genes for the Fur family regulators were also identified in this draft genome, indicating the potential anoxic metabolism of this bacterium. Therefore, it appears to be a facultative aerobic bacterium. This is consistent with the conditions of the anoxic/aerobic process from which these samples were collected, as well as the biogeographic distribution of the phylotypes from candidate phylum SBR1093. Additionally, full genes responsible for the upstream metabolism of glycolysis, such as the metabolism of glycogen/starch and cellulose, are also identified in this draft genome, but there were no genes related to the uptake of glucose or metabolism of sucrose, maltose or xylose. Therefore, this bacterium may use glycolysis only for the catabolism of self-producing sugar rather than external sugar.

Based on the genetic analysis, this bacterium may reduce nitrate via an assimilation path and nitrite via an assimilation or dissimilation path (Figure S3 in File S1), which indirectly verifies the facultative aerobic metabolism. Although most of the amino acid can be synthetized with the assimilated ammonia, no genes for asparagine synthesis were detected, implying the necessity for asparagine supplementation in the enrichment of this bacterium. This is consistent with the branched-chain amino acid ABC transporters. Additionally, only genes encoding sulfite reductase exist in this draft genome (none for sulfate reductase), indicating that this bacterium may survive in niches with relatively high redox potential rather than strictly anaerobic conditions. This is also consistent with the conditions in which the microbial community enriched this bacterium (i.e., cycling between the anoxic and aerobic tanks).

Regarding the utilization of the substrates, microbes enriched in a specific condition may adapt to this environment and interact closely with their habitat through metabolic reactions [28]. Because this bacterium is enriched in an anoxic/aerobic process and is fed with morpholine distilling wastewater, it may be involved in the metabolism of the main organics in this wastewater. However, the organics in this influent are primarily in the form of morpholine (or its derivatives), which is reported to decompose into glyoxylate and glycolate only by some species of *Mycobacterium* containing a complex of cytochrome p450, ferredoxin and ferredoxin reductase [29]. Based on the genetic analysis, there is no p450 gene in this draft genome. Therefore, this bacterium may not be active in the ring-opening of morpholine and is more likely involved in the metabolism of the intermediates of morpholine decomposition such as glycolate and glyoxylate. This is consistent with multiple genes responsible for the conversion of glycolate (gene_1890, 1891, 2884, 2885, 2887) and glyoxylate (gene_55), as well as the fusion protein responsible for the glyoxylate shunt (Table S3 in File S1, gene_720), which combines the glyoxylate with acetyl-CoA for the synthesis of malate.

### Identification of the carbon fixation pathway

**Figure 3:**
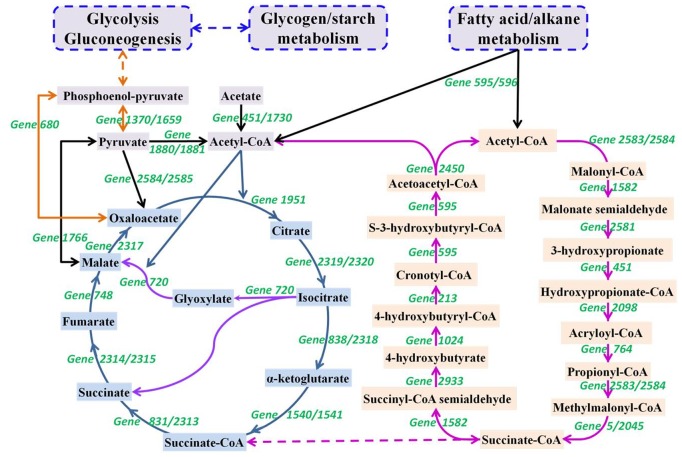
Putative metabolic pathway of SBR1093 HKSP (based on the genetic analysis). Carbon fixation with the HPHB cycle is used for the biomass synthesis via the transfer of acetyl-CoA to the citrate cycle or gluconeogenesis, and the genes responsible for each step are marked with green words. Intermediates connected with colored lines represent different metabolic pathways.

Interestingly, this draft genome possesses genes encoding phosphoenolpyruvate (PEP), acetyl-CoA and propionyl-CoA carboxylase, which catalyze carbon fixation, indicating that this bacterium might have autotrophic metabolism potential. Further comparative genetics reveal that this draft genome contains all of the 13 enzymes required for the hydroxypropionate–hydroxybutyrate (HPHB) cycle (Figure 3), a carbon fixation pathway possessed only by *Archaea* [30]. The enzymes in this draft genome share 30∼50% amino acid identity with those involved in the HPHB cycle in *Metallosphaera sedula*, a typical strain of *Archaea* that undergoes the HPHB cycle (Table 1). In this carbon fixation pathway, two molecules CO_2_ are assimilated in form of bicarbonate with the carboxylation of a bifunctional biotin-dependent acetyl-CoA/propionyl-CoA carboxylase, and the produced acetyl-CoA is transferred to the citrate cycle for the synthesis of the amino acids and fatty acids or alternatively glycogen via gluconeogenesis. For other carbon fixation pathways, some key enzymes are absent from this draft genome (Figure S4 in File S1), such as pyruvate synthase (EC:1.2.7.1) for the 3-dicarboxylate-hydroxybutyrate (DCHP) pathway, ATP-citric lyase (EC:2.3.3.8) and 2-oxoglutarate synthase (EC:1.2.7.3) for the reductive citric acid cycle pathway, NADP+ dependent formate dehydrogenase (EC:1.2.1.43) and formyltetrahydrofolate synthetase (EC:6.3.4.3) for the reductive acetyl-CoA pathway, malyl-CoA lyase (EC:4.1.3.24) for the 3-hydroxypropionate cycle pathway, and ribulose-bisphosphate carboxylase (EC:4.1.1.39) for the reductive pentose phosphate cycle pathway [31]. Therefore, SBR1093 HKSP may have autotrophic metabolism via the HPHB carbon fixation pathway, whereas the phosphoenolpyruvate carboxylase may function to maintain the balance of intermediates within the citrate cycle [32]. Additionally, enzymes employed for this carbon fixation pathway in *Archaea* are oxygen-tolerant, which is also consistent with the living conditions of this bacterium.

**Table 1:** Genes responsible for the HPHB cycle in the identified genomes and their identity with those in SBR1093.

SBR1093 HKSP	Functional enzymes	*Ac. hospitalis*		*M. sedula*		*S. tokodaii*		*N. gargensis*		*Af. felis*		*B. sp.* STM3843		*O.carboxidovorans*	
		Acc. No.	Identity	Acc. No.	Identity	Acc. No.	Identity	Acc. No.	Identity	Acc. No.	Identity	Acc. No.	Identity	Acc. No.	Identity
gene_764	ACR	58889	32%	91508	31%	78610	32%	63583	37%	19219	37%	39404	35%	31729	36%
**gene_2584**	**ACC**	**59288**	**44%**	**90248**	**45%**	**76481**	**45%**	**60794**	**51%**	**19087**	**53%**	**33415**	**51%**	**32993**	**52%**
gene_2098	HPCD	58277	45%	92065	47%	77478	44%	62785	46%	19777	41%	36654	42%	32328	35%
gene_2045	MCE	59386	40%	90738	38%	76441	44%	63084	43%	18664	42%	33379	38%	33030	42%
gene_2581	MSR	59445	41%	91505	41%	78088	43%	61994	39%	21469	44%	35006	54%	34115	43%
gene_5	MCM	59385	47%	90737	47%	76440	48%	63082	48%	//	//	36638	63%	//	//
gene_1024	HBCS	58189	29%	91107	31%	77036	30%	62874	25%	20234	36%	37419	37%	31206	37%
gene_451	HPCS	58189	48%	91435	53%	78009	52%	62874	54%	20930	57%	37438	59%	31167	55%
gene_2450	ACK	59345	41%	90755	43%	76400	41%	62339	28%	21237	59%	33768	59%	31369	58%
**gene_1582**	**MCR**	**58545**	**28%**	**91779**	**26%**	**77174**	**29%**	**60718**	**31%**	**20991**	**55%**	**38304**	**55%**	**31211**	**55%**
**gene_595**	**CCH**	**59445**	**24%**	**90500**	**29%**	**75917**	**30%**	**61994**	**23%**	**21942**	**27%**	**35636**	**29%**	**33680**	**27%**
gene_213	HBCD	59205	30%	91403	31%	77631	30%	64012	34%	19793	82%	33076	80%	32741	82%
gene_2933	SSR	59446	31%	91506	30%	78341	29%	63668	37%	19498	32%	38465	35%	32182	30%

The identified genomes are downloaded from NCBI FTP with the accession names *Acidianus hospitalis* W1 uid66875 (*Ac. hospitalis*), *Metallosphaera sedula* DSM 5348 uid58717 (*M. sedula*), *Sulfolobus tokodaii* 7 uid57807 (*S. tokodaii*), Candidatus *Nitrososphaera gargensis* Ga9 2 uid176707 (*N. gargensis*), *Afipia felis* ATCC 53690 uid179396 (*Af. felis*), Bradyrhizobium STM 3843 uid80711 (*B. sp.* STM3843), *Oligotropha carboxidovorans* OM5 uid72795 (*O. carboxidovorans*). The enzymes in bold are bifunctional enzymes in the HPHB pathway, and columns filled with//indicate that there were no hit enzymes in the genome. *M. sedula*, *S. tokodaii* and *Ac. hospitalis* are members of *Sulfolobales* and have the potential for autotrophic metabolism via the reported HPHB pathway, whereas *B. sp.* STM3843, *Af. felis* and *O. carboxidovorans* are members from *Bradyrhizobiaceae* with facultative autotrophic metabolism via an unknown carbon fixation pathway. The accession number for *Acidianus hospitalis* should have ‘YP0044’ before the presented numbers, and *Metallosphaera* should have ‘YP0011’, *Sulfolobus* ‘NP3’, *Nitrososphaera* ‘YP0068’, *Afipia* ‘ZP114’, *Bradyrhizobium* ‘ZP0943’, and *Oligotropha* ‘YP0046’.

Abbreviations for functional enzymes: Acryloyl-CoA reductase (ACR); Acetyl/propionyl-CoA carboxylase (ACC); 3-Hydroxypropionyl-CoA dehydratase (HPCD); Methylmalonyl-CoA epimerase (MCE); Malonic semialdehyde reductase (MSR); Methylmalonyl-CoA mutase (MCM); 4-Hydroxybutyrate-CoA ligase (HBCS); 3-Hydroxypropionate-CoA ligase (HPCS); Acetoacetyl-CoA β-ketothiolase (ACK); Succinyl/Malonyl-CoA reductase (MCR); Crotonyl-CoA hydratase (CCH); 4-Hydroxybutyryl-CoA dehydratase (HBCD); Succinic semialdehyde reductase (SSR).

However, microbes possessing the HPHB pathway may evolve as facultative autotrophs rather than obligate autotrophs because this carbon fixation pathway is the most energy-consuming pathway, requiring nine ATP equivalents for one pyruvate [33]. Organisms harboring this carbon fixation pathway may proliferate via a heterotrophic rather than autotrophic pathway as long as there are available organic substrates, which is the nutritional strategy of microbes [34]. For example, although many species within *Sulfolobales* are described as autotrophs or mixotrophs via this carbon fixation pathway, some strains deposited in the public culture collection could lose their autotrophic ability after continuous laboratory cultivation in nutrient-rich media [33]. According to the above discussion, SBR1093 HKSP may possess potential of both autotrophic and heterotrophic metabolisms, thus should be a mixotroph. This is consistent with the enriching conditions, as well as the habitats of strains in the same taxonomic clade. Mixotrophy has been demonstrated to be a widespread and important phenomenon in the biosphere [35], which combines the traits of autotrophs and heterotrophs to utilize both inorganic and organic resources to enable the host survive in oligotrophic or transient environments. In this study, SBR1093 HKSP is enriched in an anoxic/aerobic process, which cycled microbes continuously from feast to fast according to anoxic and aerobic conditions. The microorganisms thus adapt to the transient environment and strive for the dominant position in this microbial community.

**Figure 4:**
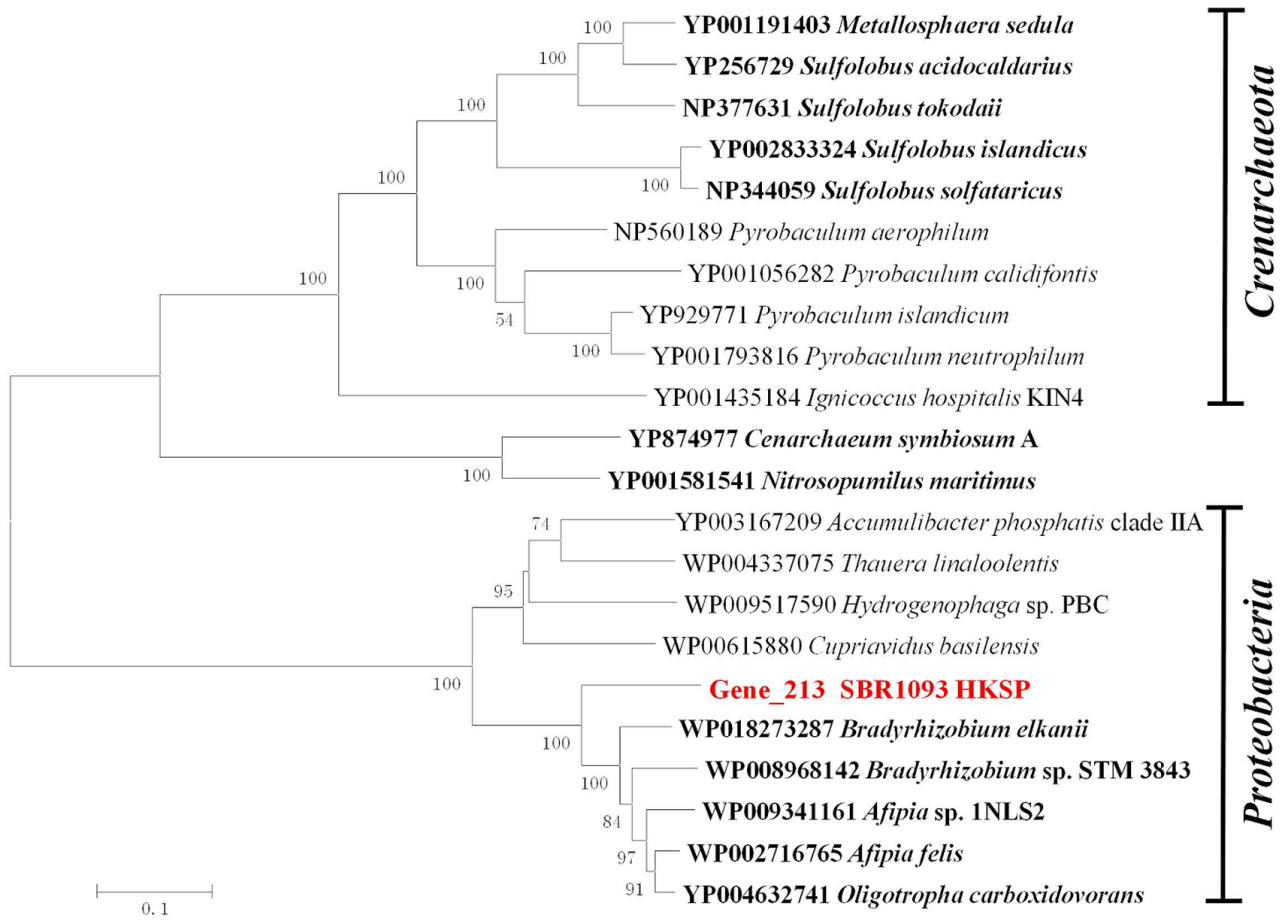
Phylogenetic tree of 4-hydroxybutyryl-CoA dehydratase proteins. The 4-hydroxybutyryl-CoA dehydratases in this draft genome and others in identified autotrophic *Achaea* were retrieved from NCBI to build the phylogenetic tree. The hosts of these marked (in bold) are suspected autotrophic microbes that undergo the HPHB cycle. The number in front of the taxonomy presents the accession number in NCBI. The tree topography and evolutionary distances are given via the neighbor-joining method with a Poisson correction. The numbers at the nodes indicate the percentage bootstrap values for the clade of this group in 1,000 replications. The scale bar represents a difference of 0.1 substitutions per site.

Additionally, in this draft genome, a key enzyme for this HPHB pathway, 4-hydroxybutyryl-CoA dehydratase (gene_213), is diverged from those in *Archaea* possessed the HPHB pathway, but clustered with facultative autotrophic bacteria such as *Bradyrhizobium* and *Afipia* within *Bradyrhizobiaceae* (Figure 4). This suggests that this gene is unlikely to be horizontally obtained across a recent domain for SBR1093. A further comparison analysis reveals that in addition to the identified autotrophic *Archaea*, all of the 13 enzymes involved in the HPHB pathway are also identified in some species within *Bradyrhizobiaceae* (Table 1), implying the wide distribution of the HPHB pathway in the biosphere and the potential importance of carbon fixation in soils and oceans. A phylogenetic analysis based on concatenated amino acid sequences, that are responsible for the transfer of succinyl-CoA to acetoacetyl-CoA and shared by DCHP & HPHB pathway, also reveals that this bacterium is closer to bacteria within *Bradyrhizobiaceae* than *Archaea* (those responsible for the transfer of crotonyl-CoA to acetoacetyl-CoA are not included because they are fusion proteins of enoyl-CoA hydratase and hydroxybutyryl-CoA dehydrogenase in bacteria but in reverse order in *Archaea*). Therefore, this bacterium may have a nutrition pattern similar to that of bacteria within *Bradyrhizobiaceae.*

Compared with the oligotrophic niches in which *Archaea* reside, *Bradyrhizobiaceae* bacteria are often found in fertile habitats rich in nutrients. Bacteria within *Bradyrhizobiaceae* often appear as heterotrophs rather than autotrophs, although most can grow chemolithoautotrophically using hydrogen, thiosulfate or sulfide as an electron donor [36]. Similarly, the bacterium SBR1093 HKSP is enriched in an industrial WWTP with an influent chemical oxygen demand (COD) concentration of up to 7,000 mg L^−1^ and ammonia concentration up to 100 mg L^−1^; thus, it may also be a facultative heterotroph or obligate mixotroph.

### Antibiotics and heavy metal resistance

It is also interesting to note that this bacterium may have multidrug resistance, such as penicillin, tetracycline, methylenomycin A, chloramphenicol and some macrolide-specific drugs, because abundance of genes associated with drug resistance and efflux transporters are identified in the draft genome. For example, 13 genes encoding a putative drug exporter in the RND superfamily may catalyze antibiotics efflux via an H+ antiport mechanism (Table S4 in File S1), and 19 genes encoding MFS transporter may have antibiotic resistance potential.

Additionally, a complete set gene associated with arsenate reduction and transport has also been identified in this draft genome, as well as 12 genes encoding the ArsR family transcriptional regulator, which may repress the expression of operons linked to stress of di- and multivalent heavy metal ions [37]. Therefore, a variety of heavy metal resistances and transporter genes can be expected in the genome, including the resistance genes for copper and mercury and transport genes for Cu^2+^ and chromate (Table S4 in File S1). The variety resistance to a variety of heavy metals is consistent with the relevant geographical distribution, ocean crust, marine sediment, contaminated soil and activated sludge.

## Conclusions

In summary, a draft genome of uncultivated bacteria from candidate phylum SBR1093 was reconstructed with the metagenome of a microbial community from a full-scale industrial wastewater treatment plant. According to the phylogenetic analysis, this bacterium belongs to clade I of candidate phylum SBR1093, which is associated with a contaminated environment and indicates the demand of organics for metabolism. Genome analysis indicates that the bacterium SBR1093 in this phylum may grow autotrophically via the HPHB cycle, a carbon fixation pathway recently found only in some genera from *Archaea*. Enzymes in this draft genome involved in carbon fixation are diverged from those in *Archaea* but share obvious homology to those found in *Bradyrhizobiaceae*. Therefore, this indicates that this bacterium may be a mixotroph. So far, all of the metabolic properties of this SBR1093 HKSP are deduced only based on the genomic analysis and comparative genomics. Further understanding of the ecological role of this candidate phylum will be obtained through its effective enrichment in the laboratory and the investigation on pure culture.

## Supporting Information

File S1 **Supporting Figures and Tables. Figure S1. Phylogenetic tree of sequences of SBR1093 and the reference sequences from representative phyla.** This phylogenetic tree is constructed with 16S rRNA gene sequences based on the maximum-likelihood method with Jukes and Cantor distances. Only bootstrap value of >50% is labeled and the phylotypes of SRB1093 are marked with red in bold. The scale bar represents 0.1 nucleotide substitutions per site. **Figure S2. Taxonomy of proteins in this SBR1093 HKSP.** Proteins are converted with the predicted gene, and performed BLASTp against NCBI nr database (released at July 18, 2013). Therefore, it is imported into Megan for taxonomic classification. **Figure S3. Putative nitrogen metabolic pathway of this SBR1093 HKSP (Adapted from KEGG). Figure S4. Suspected carbon fixation pathway possessed by this SBR1093 HKSP (Adapted from KEGG).** Columns with solid fill indicate these enzymes are identified in this draft genome, which are connected with colorful lines and arrows and each color represents a type of carbon fixation pathway. Only the red one are full filled, which represents the HPHB cycle. **Table S1. Universally occurring clusters of orthologous groups and tRNAs for amino acids identified in this draft genome. Table S2. List of ESCGs identified in this draft genome and their taxonomy. Table S3. Suspected genes in this draft genome that involving in the primary metabolism. Table S4. Suspected genes in this draft genome that involving in the antibiotics and heavy metal resistance or export.**
(PDF)
