# Supplementary material for: Evidence of Carbon Fixation Pathway in a Bacterium from Candidate Phylum SBR1093 Revealed with Genomic Analysis
Source: PLoS One. 2014 Oct 13;9(10):e109571. doi: 10.1371/journal.pone.0109571 (PMC4195664; doi:10.1371/journal.pone.0109571)
Supplement: File S1 — Supporting Figures and Tables. Figure S1. Phylogenetic tree of sequences of SBR1093 and the reference sequences from representative phyla. This phylogenetic tree is constructed with 16S rRNA gene sequences based on the maximum-likelihood method with Jukes and Cantor distances. Only bootstrap value of >50% is labeled and the phylotypes of SRB1093 are marked with red in bold. The scale bar represents 0.1 nucleotide substitutions per site. Figure S2. Taxonomy of proteins in this SBR1093 HKSP. Proteins are converted with the predicted gene, and performed BLASTp against NCBI nr database (released at July 18, 2013). Therefore, it is imported into Megan for taxonomic classification. Figure S3. Putative nitrogen metabolic pathway of this SBR1093 HKSP (Adapted from KEGG). Figure S4. Suspected carbon fixation pathway possessed by this SBR1093 HKSP (Adapted from KEGG). Columns with solid fill indicate these enzymes are identified in this draft genome, which are connected with colorful lines and arrows and each color represents a type of carbon fixation pathway. Only the red one are full filled, which represents the HPHB cycle. Table S1. Universally occurring clusters of orthologous groups and tRNAs for amino acids identified in this draft genome. Table S2. List of ESCGs identified in this draft genome and their taxonomy. Table S3. Suspected genes in this draft genome that involving in the primary metabolism. Table S4. Suspected genes in this draft genome that involving in the antibiotics and heavy metal resistance or export. (PDF) [file pone.0109571.s001.pdf]

Supplementary material for

Evidence of Carbon Fixation Pathway in a Bacterium from Candidate Phylum SBR1093 Revealed with Genomic Analysis

Submitted to ***PLOS ONE***

Zhiping Wang[1,2], Feng Guo[1], Lili Liu[1,3], Tong Zhang[1]*

[1] Environmental Biotechnology Laboratory, The University of Hong Kong, Hong Kong;

[2] School of Environmental Science and Engineering, Shanghai Jiao Tong University, Shanghai;

[3] State Environmental Protection Key Laboratory of Environmental Risk Assessment and Control on Chemical Process, East China University of Science and Technology, Shanghai, China

*Corresponding author: Tong Zhang, Environmental Biotechnology Laboratory, The University of Hong Kong, Hong Kong. Tel. 852-2857-8551, E-mail: zhangt@hku.hk

Total pages: 17

Figures: 4

Tables: 4

Figure S1 Phylogenetic tree of sequences of SBR1093 and the reference sequences from representative phyla. This phylogenetic tree is constructed with 16S rRNA gene sequences based on the maximum-likelihood method with Jukes and Cantor distances. Only bootstrap value of > 50% is labeled and the phylotypes of SRB1093 are marked with red in bold. The scale bar represents 0.1 nucleotide substitutions per site.

Figure S2 Taxonomy of proteins in this SBR1093 HKSP. Proteins are converted with the predicted gene, and performed BLASTp against NCBI nr database (released at July 18, 2013). Therefore, it is imported into Megan for taxonomic classification.

Figure S3 Putative nitrogen metabolic pathway of this SBR1093 HKSP (Adapted from KEGG).

Figure S4 Suspected carbon fixation pathway possessed by this SBR1093 HKSP (Adapted from KEGG). Columns with solid fill indicate these enzymes are identified in this draft genome, which are connected with colorful lines and arrows and each color represents a type of carbon fixation pathway. Only the red one are full filled, which represents the HPHB cycle.

Table S1 Universally occurring clusters of orthologous groups and tRNAs for amino acids identified in this draft genome.

Table S2 List of ESCGs identified in this draft genome and their taxonomy.

Table S3 Suspected genes in this draft genome that involving in the primary metabolism.

Table S4 Suspected genes in this draft genome that involving in the antibiotics and heavy metal resistance or export.

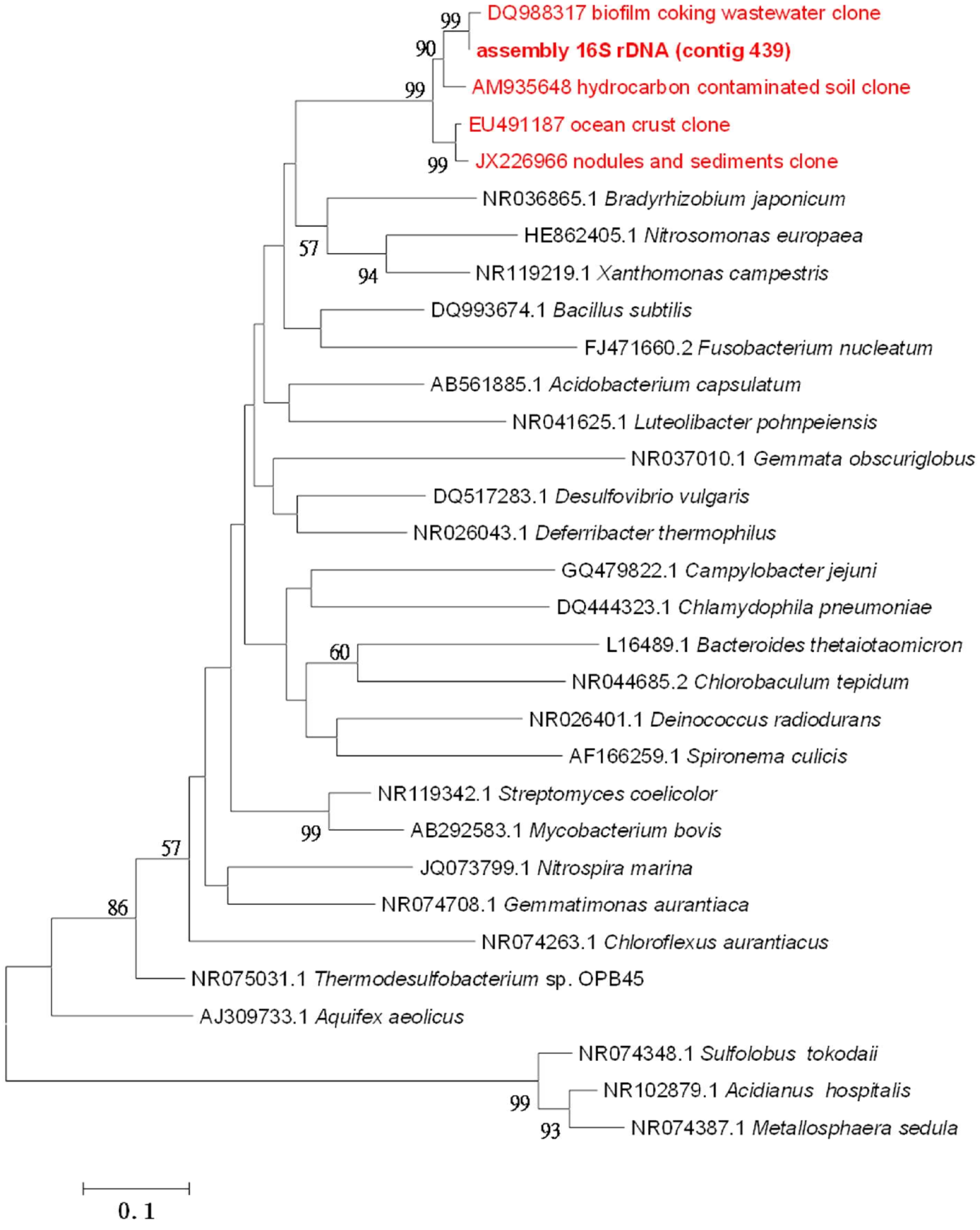

Figure S1 Phylogenetic tree of sequences of SBR1093 and the reference sequences from representative phyla. This phylogenetic tree is constructed with 16S rRNA gene sequences based on the maximum-likelihood method with Jukes and Cantor distances. Only bootstrap value of > 50% is labeled and the phylotypes of SRB1093 are marked with red in bold. The scale bar represents 0.1 nucleotide substitutions per site.

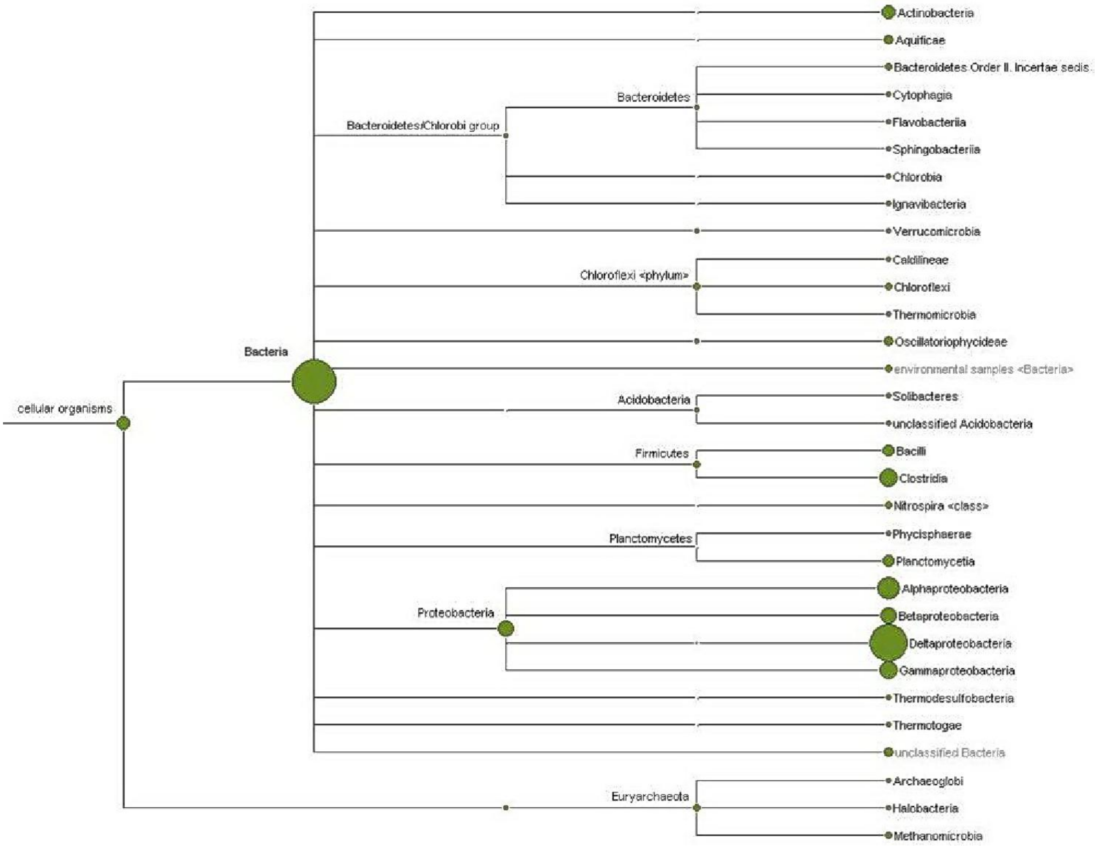

Figure S2 Taxonomy of proteins in this SBR1093 HKSP. Proteins are converted with the predicted gene, and performed BLASTp against NCBI nr database (released at July 18, 2013). Therefore, the results can be imported into Megan for taxonomic classification.

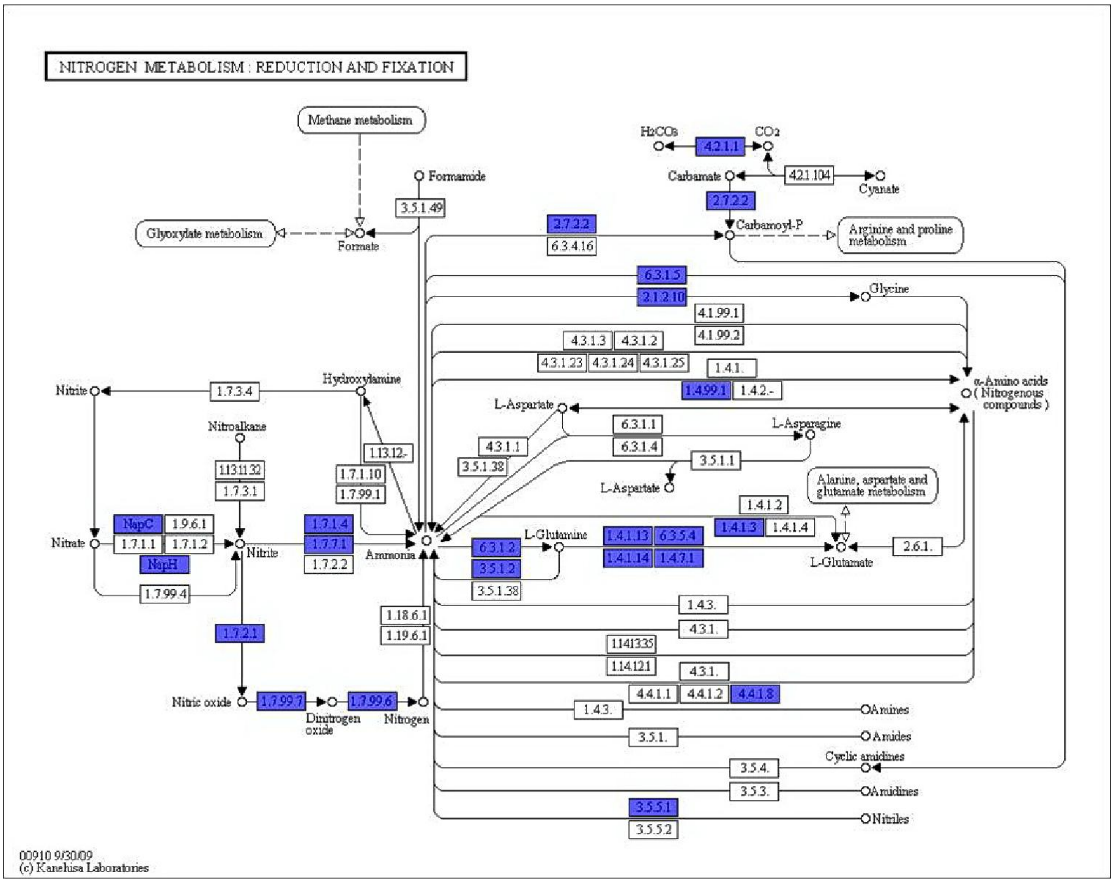

Figure S3 Putative nitrogen metabolic pathway of this SBR1093 HKSP (Adapted from KEGG).

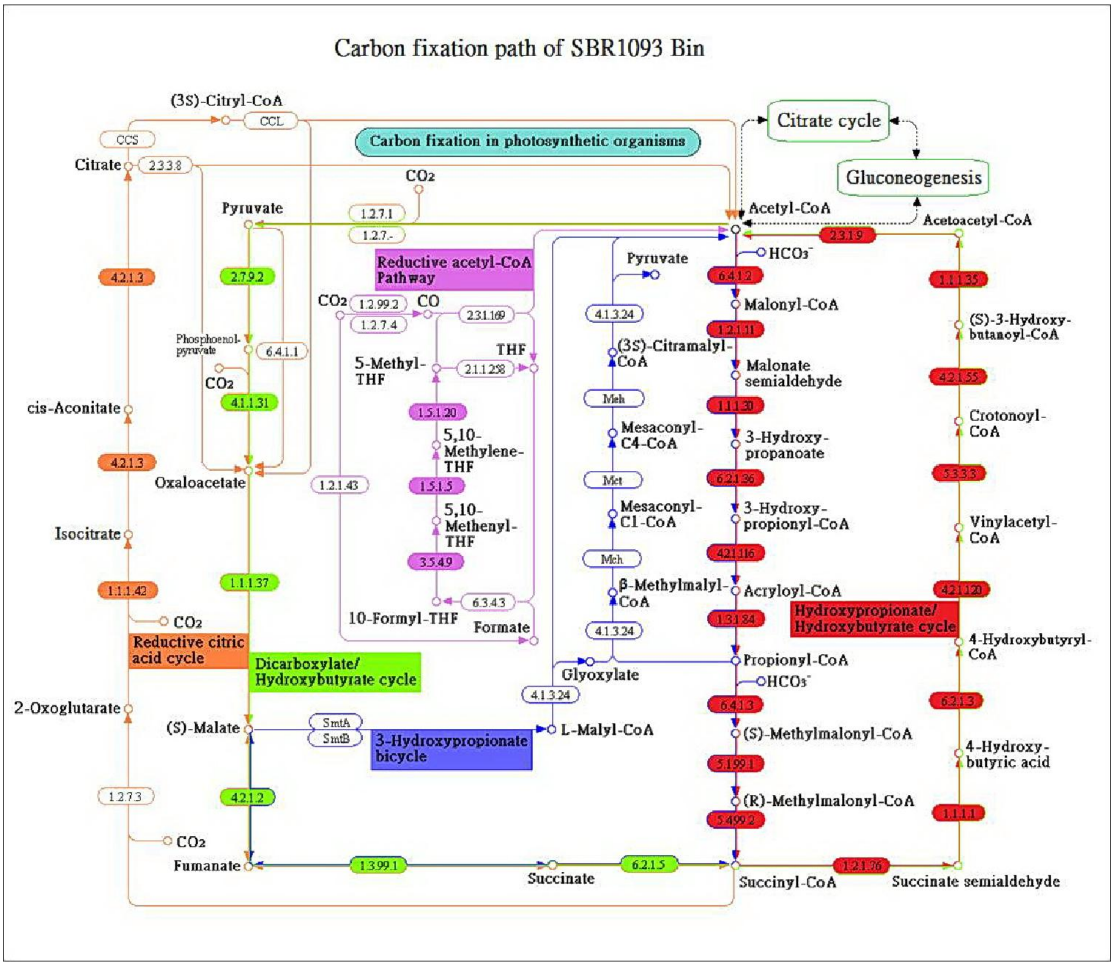

Figure S4 Suspected carbon fixation pathway possessed by this SBR1093 HKSP (Adapted from KEGG). Columns with solid fill indicate these enzymes are identified in this draft genome, which are connected with colorful lines and arrows and each color represents a type of carbon fixation pathway. Only the HPHB cycle marked with red one are complete, thus suggesting the autotroph potential of this bacterium

Table S1 40 universally occurring clusters of orthologous groups and tRNAs for all 20 amino acids.

40 Universal COGs		
Gene_id	COG No.	COG description
gene_316	COG0012	Predicted GTPase, probable translation factor
gene_817	COG0016	Phenylalanyl-tRNA synthetase alpha subunit
gene_856	COG0018	Arginyl-tRNA synthetase
gene_1508	COG0048	Ribosomal protein S12
gene_1507	COG0049	Ribosomal protein S7
gene_546	COG0052	Ribosomal protein S2
gene_2384	COG0080	Ribosomal protein L11
gene_2385	COG0081	Ribosomal protein L1
gene_2388	COG0085	DNA-directed RNA polymerase, beta subunit
gene_2964	COG0087	Ribosomal protein L3
gene_2965	COG0088	Ribosomal protein L4
gene_2967	COG0090	Ribosomal protein L2
gene_2969	COG0091	Ribosomal protein L22
gene_2970	COG0092	Ribosomal protein S3
gene_2974	COG0093	Ribosomal protein L14
gene_2976	COG0094	Ribosomal protein L5
gene_2978	COG0096	Ribosomal protein S8
gene_2979	COG0097	Ribosomal protein L6P/L9E
gene_2981	COG0098	Ribosomal protein S5
gene_2987	COG0099	Ribosomal protein S13
gene_2988	COG0100	Ribosomal protein S11
gene_2883	COG0102	Ribosomal protein L13
gene_2882	COG0103	Ribosomal protein S9
gene_757	COG0124	Histidyl-tRNA synthetase
gene_552	COG0172	Seryl-tRNA synthetase
gene_1783	COG0184	Ribosomal protein S15P/S13E
gene_2968	COG0185	Ribosomal protein S19
gene_2973	COG0186	Ribosomal protein S17
gene_2971	COG0197	Ribosomal protein L16/L10E

gene_2982	COG0200	Ribosomal protein L15
gene_2983	COG0201	Preprotein translocase subunit SecY
gene_2990	COG0202	DNA-directed RNA polymerase, alpha subunit
gene_1195	COG0215	Cysteinyl-tRNA synthetase
gene_2980	COG0256	Ribosomal protein L18
gene_2596	COG0495	Leucyl-tRNA synthetase
gene_2989	COG0522	Ribosomal protein S4 and related proteins
gene_2390	COG0525	Valyl-tRNA synthetase
gene_887	COG0533	Metal-dependent proteases
gene_557	COG0541	Signal recognition particle GTPase
gene_1079	COG0552	Signal recognition particle GTPase
20 tRNAs for amino acid		
gene_2580	COG0008	glutamyl-tRNA synthetase
gene_2294	COG0008	glutaminyl-tRNA synthetase
gene_2399	COG0013	alanyl-tRNA synthetase
gene_817	COG0016	phenylalanyl-tRNA synthetase alpha chain
gene_856	COG0018	arginyl-tRNA synthetase
gene_1887	COG0060	isoleucyl-tRNA synthetase
gene_2682	COG0072	phenylalanyl-tRNA synthetase beta chain
gene_757	COG0124	histidyl-tRNA synthetase
gene_1343	COG0143	methionyl-tRNA synthetase
gene_2901	COG0162	tyrosyl-tRNA synthetase
gene_552	COG0172	seryl-tRNA synthetase
gene_1535	COG0173	aspartyl-tRNA synthetase
gene_2943	COG0180	tryptophanyl-tRNA synthetase
gene_1195	COG0215	cysteinyl-tRNA synthetase
gene_935	COG0441	threonyl-tRNA synthetase
gene_1257	COG0442	prolyl-tRNA synthetase
gene_3062	COG0495	leucyl-tRNA synthetase
gene_2390	COG0525	valyl-tRNA synthetase
gene_2832	COG0751	glycyl-tRNA synthetase beta chain
gene_2834	COG0752	glycyl-tRNA synthetase alpha chain
gene_2694	COG1190	lysyl-tRNA synthetase, class II

Table S2 List of ESCGs identified in this draft genome*

**gene_2689**	**PF00162.14**	***Thaumarchaeota***		gene_757	TIGR00442	*Proteobacteria*
**gene_1216**	**PF00162.14**	***NA***		gene_1535	TIGR00459	*Firmicutes*
gene_2966	PF00276.15	*Proteobacteria*		gene_632	TIGR00460	*Proteobacteria*
gene_2976	PF00281.14	*Proteobacteria*		gene_817	TIGR00468	*Proteobacteria*
gene_2964	PF00297.17	*Proteobacteria*		gene_2682	TIGR00472	*NA*
gene_2979	PF00347.18	*Proteobacteria*		gene_1788	TIGR00487	*Proteobacteria*
gene_2973	PF00366.15	*Proteobacteria*		gene_549	TIGR00496	*Firmicutes*
gene_2882	PF00380.14	*Firmicutes*		gene_1251	TIGR00575	*Proteobacteria*
gene_2978	PF00410.14	*Proteobacteria*		gene_2022	TIGR00663	*NA*
gene_2988	PF00411.14	*Proteobacteria*		gene_1214	TIGR00810	*NA*
gene_2987	PF00416.17	*Proteobacteria*		gene_2387	TIGR00855	*Proteobacteria*
gene_2386	PF00466.15	*Proteobacteria*		gene_2383	TIGR00922	*Proteobacteria*
gene_2965	PF00573.17	*Proteobacteria*		gene_1783	TIGR00952	*Proteobacteria*
**gene_856**	**PF00750.14**	***Firmicutes***		gene_557	TIGR00959	*Firmicutes*
**gene_2598**	**PF00750.14**	***Proteobacteria***		gene_664	TIGR00963	*NA*
gene_2307	PF01025.14	*Proteobacteria*		gene_2382	TIGR00964	*Proteobacteria*
gene_1060	PF01795.14	*NA*		gene_2983	TIGR00967	*Proteobacteria*
gene_933	TIGR00001	*NA*		gene_1508	TIGR00981	*Proteobacteria*
gene_558	TIGR00002	*Firmicutes*		gene_2970	TIGR01009	*Proteobacteria*
gene_449	TIGR00009	*Spirochaetes*		gene_546	TIGR01011	*Firmicutes*
gene_2972	TIGR00012	*Proteobacteria*		gene_2989	TIGR01017	*Proteobacteria*
gene_2106	TIGR00019	*NA*		gene_2981	TIGR01021	*Proteobacteria*
gene_272	TIGR00029	*Actinobacteria*		gene_562	TIGR01024	*Firmicutes*
gene_2628	TIGR00043	*Firmicutes*		gene_1507	TIGR01029	*Proteobacteria*
gene_2991	TIGR00059	*Proteobacteria*		**gene_2570_1**	**TIGR01030**	***Deinococcus***
gene_2980	TIGR00060	*Proteobacteria*		gene_2326	TIGR01031	*NA*
gene_1926	TIGR00061	*Proteobacteria*		gene_932	TIGR01032	*NA*
gene_1925	TIGR00062	*Proteobacteria*		gene_2969	TIGR01044	*Proteobacteria*
gene_1079	TIGR00064	*NA*		gene_2963	TIGR01049	*Proteobacteria*
gene_1786	TIGR00082	*Proteobacteria*		gene_2968	TIGR01050	*Proteobacteria*
gene_164	TIGR00086	*Firmicutes*		gene_814	TIGR01059	*NA*
gene_316	TIGR00092	*NA*		gene_2883	TIGR01066	*Firmicutes*
gene_1149	TIGR00115	*Proteobacteria*		gene_2974	TIGR01067	*Proteobacteria*

gene_547	TIGR00116	*Firmicutes*		gene_2982	TIGR01071	*Proteobacteria*
gene_2582	TIGR00152	*Deinococcus*		gene_2975	TIGR01079	*Proteobacteria*
gene_403	TIGR00158	*Spirochaetes*		gene_2971	TIGR01164	*Proteobacteria*
gene_402	TIGR00165	*Spirochaetes*		gene_2385	TIGR01169	*Proteobacteria*
gene_401	TIGR00166	*NA*		gene_2967	TIGR01171	*Proteobacteria*
gene_934	TIGR00168	*NA*		gene_1429	TIGR01391	*NA*
gene_2901	TIGR00234	*NA*		gene_2554	TIGR01393	*Deinococcus*
gene_1957	TIGR00337	*NA*		gene_2384	TIGR01632	*Proteobacteria*
gene_2399	TIGR00344	*Proteobacteria*		gene_1789	TIGR01953	*Proteobacteria*
gene_2021	TIGR00362	*NA*		gene_1189	TIGR02012	*Proteobacteria*
gene_1887	TIGR00392	*Proteobacteria*		gene_2388	TIGR02013	*Proteobacteria*
gene_2598	TIGR00396	*Proteobacteria*		gene_2990	TIGR02027	*Proteobacteria*
gene_181	TIGR00409	*Proteobacteria*		**gene_2311**	**TIGR02350**	***Proteobacteria***
gene_552	TIGR00414	*Firmicutes*		**gene_2306**	**TIGR02350**	***Proteobacteria***
gene_935	TIGR00418	*NA*		gene_2389	TIGR02386	*Proteobacteria*
gene_1923	TIGR00420	*NA*		gene_2182	TIGR02397	*Proteobacteria*
gene_2390	TIGR00422	*Proteobacteria*		gene_2679	TIGR02432	*NA*
gene_1195	TIGR00435	*Proteobacteria*		gene_2426	TIGR02729	*Proteobacteria*
**gene_1533**	**TIGR00436**	***Firmicutes***		gene_1760	TIGR03263	*Proteobacteria*
**gene_3015**	**TIGR00436**	***NA***		gene_3015	TIGR03594	*NA*

* Since there is no ESCGs set for this phylum, these ESCGs are identified based on HMM search with the relative taxonomy. Those suspected duplicates are marked with red bold, which may not contaminations from other bacterium according to the genetic analysis. Gene _2570_1 represents the ESCG TIGR01030, encoding the ribosomal protein L34 with 44 amino acids, which is really existed on contig_1317 but omitted by MetaGeneMark.

Table S3 Genes in this draft genome that involving in the primary metabolism.

Genes involving in Glycolysis / Gluconeogenesis pathway			
Gene_id	KO No.	EC number	KO description
gene_365	K01835	5.4.2.2	phosphoglucomutase
gene_910	K01810	5.3.1.9	glucose-6-phosphate isomerase
gene_9	K03841	3.1.3.11	fructose-1,6-bisphosphatase
gene_2075	K00850	2.7.1.11	6-phosphofructokinase
gene_2690	K01623	4.1.2.13	fructose-bisphosphate aldolase
gene_1215	K01803	5.3.1.1	triosephosphate isomerase (TIM)
gene_2688	K00134	1.2.1.12	glyceraldehyde 3-phosphate dehydrogenase
gene_1216	K00927	2.7.2.3	phosphoglycerate kinase
gene_2689	K00927	2.7.2.3	phosphoglycerate kinase
gene_857	K15634	5.4.2.1	phosphoglycerate mutase
gene_1820	K01689	4.2.1.11	enolase
gene_1370	K00873	2.7.1.40	pyruvate kinase
gene_1659	K00873	2.7.1.40	pyruvate kinase
gene_1157	K01792	5.1.3.15	glucose-6-phosphate 1-epimerase
gene_2677	K00845	2.7.1.2	glucokinase
gene_914	K00886	2.7.1.63	polyphosphate glucokinase
gene_910	K13810	5.3.1.9	transaldolase / glucose-6-phosphate isomerase
gene_911	K13810	5.3.1.9	transaldolase / glucose-6-phosphate isomerase
Genes involving in citrate cycle pathway			
gene_1951	K01647	2.3.3.1	citrate synthase
gene_2319	K01681	4.2.1.3	aconitate hydratase 1
gene_2320	K01681	4.2.1.3	aconitate hydratase 1
gene_838	K00030	1.1.1.41	isocitrate dehydrogenase (NAD^+)
gene_2318	K00031	1.1.1.41	isocitrate dehydrogenase
gene_1540	K00164	1.2.4.2	2-oxoglutarate dehydrogenase E1 component
gene_1541	K00658	2.3.1.61	2-oxoglutarate dehydrogenase E2 component
gene_831	K01902	6.2.1.5	succinyl-CoA synthetase alpha subunit

gene_2313	K01903	6.2.1.5	succinyl-CoA synthetase beta subunit
gene_2655	K00245	1.3.99.1	fumarate reductase iron-sulfur protein
gene_2315	K00239	1.3.99.1	succinate dehydrogenase flavoprotein subunit
gene_2314	K00240	1.3.99.1	succinate dehydrogenase iron-sulfur protein
gene_748	K01679	4.2.1.2	fumarate hydratase, class II
gene_2317	K00024	1.1.1.37	malate dehydrogenase
gene_720*	K01637	4.1.3.1	isocitrate lyase
gene_720*	K01637	2.3.3.9	Malate synthase
Genes involving in oxidative phosphorylation			
gene_1712	K00329	EC:1.6.5.3	NADH dehydrogenase
gene_2371	K00329	EC:1.6.5.3	NADH dehydrogenase
gene_1349	K00330	EC:1.6.5.3	NADH dehydrogenase I subunit A
gene_1350	K00331	EC:1.6.5.3	NADH dehydrogenase I subunit B
gene_1351	K00332	EC:1.6.5.3	NADH dehydrogenase I subunit C
gene_1352	K00333	EC:1.6.5.3	NADH dehydrogenase I subunit D
gene_598	K00334	EC:1.6.5.3	NADH dehydrogenase I subunit E
gene_736	K00335	EC:1.6.5.3	NADH dehydrogenase I subunit F
gene_811	K00336	EC:1.6.5.3	NADH dehydrogenase I subunit G
gene_812	K00337	EC:1.6.5.3	NADH dehydrogenase I subunit H
gene_505	K00338	EC:1.6.5.3	NADH dehydrogenase I subunit I
gene_1042	K00339	EC:1.6.5.3	NADH dehydrogenase I subunit J
gene_1041	K00340	EC:1.6.5.3	NADH dehydrogenase I subunit K
gene_1040	K00341	EC:1.6.5.3	NADH dehydrogenase I subunit L
gene_1682	K00342	EC:1.6.5.3	NADH dehydrogenase I subunit M
gene_1358	K00343	EC:1.6.5.3	NADH dehydrogenase I subunit N
gene_2151	K00356	EC:1.6.99.3	NADH dehydrogenase
gene_2660	K00356	EC:1.6.99.3	NADH dehydrogenase
gene_732	K03885	EC:1.6.99.3	NADH dehydrogenase
gene_774	K03885	EC:1.6.99.3	NADH dehydrogenase
gene_1270	K03885	EC:1.6.99.3	NADH dehydrogenase
gene_1471	K03885	EC:1.6.99.3	NADH dehydrogenase
gene_505	K03941	EC:1.6.99.3	NADH dehydrogenase (ubiquinone) Fe-S protein 8
gene_2315	K00239	EC:1.3.99.1	succinate dehydrogenase flavoprotein

			subunit
gene_2314	K00240	EC:1.3.99.1	succinate dehydrogenase iron-sulfur protein
gene_2655	K00245	EC:1.3.99.1	fumarate reductase iron-sulfur protein
gene_773	K00412	EC:1.10.2.2	ubiquinol-cytochrome c reductase cytochrome b subunit
gene_333	K00404	EC:1.9.3.1	cb-type cytochrome c oxidase subunit I
gene_2498	K00404	EC:1.9.3.1	cb-type cytochrome c oxidase subunit I
gene_1593	K00405	EC:1.9.3.1	cb-type cytochrome c oxidase subunit II
gene_2499	K00405	EC:1.9.3.1	cb-type cytochrome c oxidase subunit II
gene_2500	K00405	EC:1.9.3.1	cb-type cytochrome c oxidase subunit II
gene_335	K00406	EC:1.9.3.1	cb-type cytochrome c oxidase subunit III
gene_1593	K00406	EC:1.9.3.1	cb-type cytochrome c oxidase subunit III
gene_2184	K00406	EC:1.9.3.1	cb-type cytochrome c oxidase subunit III
gene_919	K02266	EC:1.9.3.1	cytochrome c oxidase subunit VIa
gene_768	K02274	EC:1.9.3.1	cytochrome c oxidase subunit I
gene_1555	K02274	EC:1.9.3.1	cytochrome c oxidase subunit I
gene_1776	K02274	EC:1.9.3.1	cytochrome c oxidase subunit I
gene_767	K02275	EC:1.9.3.1	cytochrome c oxidase subunit II
gene_1556	K02275	EC:1.9.3.1	cytochrome c oxidase subunit II
gene_2500	K02275	EC:1.9.3.1	cytochrome c oxidase subunit II
gene_769	K02276	EC:1.9.3.1	cytochrome c oxidase subunit III
gene_1554	K02276	EC:1.9.3.1	cytochrome c oxidase subunit III
gene_1553	K02277	EC:1.9.3.1	cytochrome c oxidase subunit IV
gene_37	K02108	EC:3.6.3.14	F-type H^+-transporting ATPase subunit a
gene_570	K02109	EC:3.6.3.14	F-type H^+-transporting ATPase subunit b
gene_571	K02109	EC:3.6.3.14	F-type H^+-transporting ATPase subunit b
gene_36	K02110	EC:3.6.3.14	F-type H^+-transporting ATPase subunit c
gene_568	K02111	EC:3.6.3.14	F-type H^+-transporting ATPase subunit alpha
gene_566	K02112	EC:3.6.3.14	F-type H^+-transporting ATPase subunit beta
gene_569	K02113	EC:3.6.3.14	F-type H^+-transporting ATPase subunit delta
gene_565	K02114	EC:3.6.3.14	F-type H^+-transporting ATPase subunit epsilon
gene_567	K02115	EC:3.6.3.14	F-type H^+-transporting ATPase subunit

			gamma
gene_400	K01507	EC:3.6.1.1	inorganic pyrophosphatase
gene_3046	K01507	EC:3.6.1.1	inorganic pyrophosphatase
gene_2613	K00937	EC:2.7.4.1	polyphosphate kinase
gene_2646	K00937	EC:2.7.4.1	polyphosphate kinase

* Gene_720 represents a fusion gene of PF01274 and PF00463, responsible for malate synthase and isocitrate lyase, which are expressed synchronously to complement the glyoxylate shunt.

Table S4 Genes in this draft genome with putative antibiotics and heavy metal resistance potential.

Genes	putative type for antibiotics resistance
204	antibiotic transport system ATP-binding protein
252	antibiotic transport system ATP-binding protein
309	antibiotic transport system ATP-binding protein
1539	antibiotic transport system ATP-binding protein
1963	antibiotic transport system ATP-binding protein
1964	antibiotic transport system ATP-binding protein
1965	antibiotic transport system ATP-binding protein
205	antibiotic transport system permease protein
206	antibiotic transport system permease protein
310	antibiotic transport system permease protein
1962	antibiotic transport system permease protein
2610	beta-lactamase
2611	beta-lactamase
891	MarR family transcriptional regulator
1154	MarR family transcriptional regulator
3030	MarR family transcriptional regulator
674	MFS transporter, DHA1 family, multidrug resistance protein
1751	MFS transporter, DHA1 family, multidrug resistance protein
2692	MFS transporter, DHA1 family, multidrug resistance protein
2374	MFS transporter, DHA1 family, tetracycline resistance protein
2954	MFS transporter, DHA1 family, tetracycline resistance protein
1374	MFS transporter, DHA2 family, methyl viologen resistance protein SmvA
2509	MFS transporter, DHA2 family, methylenomycin A resistance protein
674	MFS transporter, DHA2 family, multidrug resistance protein B
1375	MFS transporter, DHA2 family, multidrug resistance protein B
2533	MFS transporter, DHA2 family, multidrug resistance protein B
1710	multidrug resistance outer membrane protein OprJ
1711	multidrug resistance outer membrane protein OprJ
1743	multidrug resistance outer membrane protein OprJ
219	multidrug resistance protein A
778	multidrug resistance protein A

1940	multidrug resistance protein A
1901	penicillin-binding protein 1A
1571	penicillin-binding protein 1C
1560	penicillin-binding protein 2
2164	penicillin-insensitive murein endopeptidase
16	putative drug exporter of the RND superfamily
464	putative drug exporter of the RND superfamily
801	putative drug exporter of the RND superfamily
1114	putative drug exporter of the RND superfamily
1566	putative drug exporter of the RND superfamily
1631	putative drug exporter of the RND superfamily
1947	putative drug exporter of the RND superfamily
2007	putative drug exporter of the RND superfamily
2269	putative drug exporter of the RND superfamily
2496	putative drug exporter of the RND superfamily
2671	putative drug exporter of the RND superfamily
1261	putative multidrug efflux transporter MdtA
2070	putative multidrug efflux transporter MdtA
Genes	putative type for heavy metal resistance
195	arsenate reductase
196	arsenate reductase
2256	arsenate reductase
2523	arsenical resistance protein ArsH
2014	arsenite methyltransferase
318	arsenite-transporting ATPase
1247	arsenite-transporting ATPase
1384	arsenite-transporting ATPase
2134	arsenite-transporting ATPase
1662	chromate transporter
1775	Cu(I)/Ag(I) efflux system membrane protein CusA
1602	Cu(I)/Ag(I) efflux system membrane protein CusB
1774	Cu(I)/Ag(I) efflux system membrane protein CusB
1710	Cu(I)/Ag(I) efflux system outer membrane protein CusC
1743	Cu(I)/Ag(I) efflux system outer membrane protein CusC
1939	Cu(I)/Ag(I) efflux system outer membrane protein CusC
330	Cu^{2+}-exporting ATPase

331	Cu^{2+}-exporting ATPase
1603	Cu^{2+}-exporting ATPase
2227	Cu^{2+}-exporting ATPase
2502	Cu^{2+}-exporting ATPase
2503	Cu^{2+}-exporting ATPase
1593	putative copper resistance protein D
1272	suppressor for copper-sensitivity B
299	heavy-metal exporter, HME family
1711	heavy-metal exporter, HME family
1773	heavy-metal exporter, HME family
1937	mercuric reductase
